# N-acetyl cysteine administration affects cerebral blood flow as measured by arterial spin labeling MRI in patients with multiple sclerosis

**DOI:** 10.1016/j.heliyon.2021.e07615

**Published:** 2021-07-16

**Authors:** Shiva Shahrampour, Justin Heholt, Andrew Wang, Faezeh Vedaei, Feroze B. Mohamed, Mahdi Alizadeh, Ze Wang, George Zabrecky, Nancy Wintering, Anthony J. Bazzan, Thomas P. Leist, Daniel A. Monti, Andrew B. Newberg

**Affiliations:** a Department of Radiology, Thomas Jefferson University, Philadelphia, PA, USA; b Charles E. Schmidt College of Medicine, Marcus Institute of Integrative Health at FAU Medicine, Florida Atlantic University, Boca Raton, FL USA; c Department of Radiology, Department of Diagnostic Radiology and Nuclear Medicine, University of Maryland School of Medicine, Baltimore, MD, USA; d Department of Integrative Medicine and Nutritional Sciences, Marcus Institute of Integrative Health, Thomas Jefferson University, Philadelphia, PA, USA; e Department of Neurology, Thomas Jefferson University, Philadelphia, PA, USA

**Keywords:** N-acetyl cysteine, NAC, Antioxidant, Multiple sclerosis, Arterial spin labeling MRI, Cerebral blood flow, Cognition

## Abstract

**Background:**

The purpose of this study was to explore if administration of N-acetyl-cysteine (NAC) in patients with multiple sclerosis (MS) resulted in altered cerebral blood flow (CBF) based on Arterial Spin Labeling (ASL) magnetic resonance imaging (MRI).

**Methods:**

Twenty-three patients with mild to moderate MS, (17 relapsing remitting and 6 primary progressive) were randomized to either NAC plus standard of care (N = 11), or standard of care only (N = 12). The experimental group received NAC intravenously (50 mg/kg) once per week and orally (500mg 2x/day) the other six days. Patients in both groups were evaluated initially and after 2 months (of receiving the NAC or waitlist control) with ASL MRI to measure CBF. Clinical symptom questionnaires were also completed at both time points.

**Results:**

The CBF data showed significant differences in several brain regions including the pons, midbrain, left temporal and frontal lobe, left thalamus, right middle frontal lobe and right temporal/hippocampus (p < 0.001) in the MS group after treatment with NAC, when compared to the control group. Self-reported scores related to cognition and attention were also significantly improved in the NAC group as compared to the control group.

**Conclusions:**

The results of this study suggest that NAC administration alters resting CBF in MS patients, and this is associated with qualitative improvements in cognition and attention. Given these findings, large scale efficacy studies will be of value to determine the potential clinical impact of NAC over the course of illness in patients with MS, as well as the most effective dosages and differential effects across subpopulations.

## 1 Introduction

Multiple sclerosis (MS) is a chronic neurological disorder characterized by focal lesions of autoimmune-mediated demyelination and inflammation in the CNS. The course and clinical presentation of MS is heterogeneous, complex, and incurable. Patients may experience a range of neurologic and cognitive impairments that manifest through acute episodes of reversible neurological damage followed by progressive neurodegeneration while other patients may experience a progressive neurodegeneration from onset [1]. Although MS is an autoimmune mediated disease, there is an association between oxidative damage and CNS injury [2].

Oxidative stress is commonly characterized by a redox imbalance leading to the accumulation of oxidative species and/or a depletion of antioxidants [3]. In MS, autoimmune induced oxidative damage contributes to a variety of biological disturbances including the disruption of the blood brain barrier, the loss of oligodendrocytes, and mitochondrial dysfunction in neurons [4, 5]. These disruptions play a prominent role in the pathophysiology of MS. The brain is particularly vulnerable to oxidative stress due to its low antioxidant content, such as glutathione, and its high rate of metabolic activity [6]. Glutathione (GSH) is implicated in reducing oxidative radicals through glutathione peroxidase while also governing the gene expression of inflammatory pathways [3, 7]. The role of oxidative stress is further substantiated by the depletion of glutathione stores as seen in both mouse models and MS patient MRI scans [4, 8, 9]. Therefore, we are investigating interventions aimed at restoring the redox imbalance seen in MS in order to mitigate the effects of chronic oxidative stress in the CNS. These interventions, if effective, may improve brain function and restore neuronal function [10].

It is also known that there is neurovascular coupling that helps to ensure that functioning neurons receive sufficient blood flow to maintain their need for oxygen and glucose [11]. Evidence suggests that this coupling is preserved in MS patients [12]. However, in a variety of neurological disorders, there is a decrease in cerebral blood flow mediated either directly by the disease process affecting the microvasculature or secondary to neuronal dysfunction [13]. Similarly, MS has been shown to have decreased perfusion to certain areas of the brain [14, 15, 16, 17] which is the result of neuronal dysfunction. Such dysfunction is the result of oxidative injury in cortical neurons or retrograde neurodegeneration due to axonal injury from demyelination in the white matter [18]. Compensatory functional adaptations might also account for MS-related changes in brain perfusion and activity [19]. However, if the oxidative stress on injured neurons can be reduced, the decreased reactive oxygen species should allow neurons to restore their function and regulation of their associated microvasculature. Thus, improved neuronal function should be associated with increased cerebral blood flow that can be measured using various functional neuroimaging tools such as MRI.

The existing treatments utilized for MS focus on mitigating the immune response through methods such as dampening lymphocytic activation and infiltration. While these treatments are effective in attenuating the immune response, they fall short in preventing disease progression. Several integrative approaches for reducing psychological stress have been shown to help reduce the development of new brain lesions and also attenuated the accumulation of brain atrophy [20]. Similarly, lower overall stress appears to reduce future grey matter atrophy in MS patients [21]. In spite of these approaches, many MS patients not only continue to suffer cognitive and functional debilitation through recurrent attacks, but they are also susceptible to chronic inflammation [22, 23]. It has been shown that patients on interferon-ß1 still experience a consistent reduction in neuronal metabolic activity [24]. Finding a therapeutic target that ameliorates oxidative stress in MS patients may serve as a beneficial adjuvant therapy.

N-acetylcysteine (NAC), an antioxidant and glutathione (GSH) precursor, is promising in this regard. NAC has been shown to increase glutathione levels in not only hepatocytes but also in mice brains [25, 26, 27]. Additional data involving NAC and experimental autoimmune encephalitis (EAE) rodent models have shown positive results in attenuating inflammatory cytokine release and oxidative stress while improving clinical signs in rats [28, 29]. NAC has also been shown to cross the blood brain barrier to improve neuronal function through other various pathways [30]. In a previously published article on the same subjects, but with concomitantly obtained fluorodeoxyglucose (FDG) positron emission tomography (PET), we showed that there was increased cerebral glucose metabolism in specific subregions of the temporal and frontal lobes in MS patients when administering NAC to patients with PPMS and RRMS [31]. Thus, NAC may present as a promising therapy for mitigating oxidative stress within patients with MS.

Magnetic resonance imaging (MRI) is used primarily to assess MS through quantifying lesions and analyzing brain atrophy. T2-weighted and FLAIR sequences often show enhancements signifying the presence of demyelinating and inflammatory lesions [32]. MRI is also used to monitor treatment through a combined measure of characterizing new lesions, brain atrophy, and documenting recurrent episodes as well as patient disability [33]. This analysis, however, does not sufficiently characterize oxidative stress MS patients accrue over time. It has been discussed that reactive oxygen species are capable of significantly altering cerebral blood flow within the brain, which cannot be visualized on conventional MRI methods [13]. Using magnetically labeled arterial blood as the endogenous tracer, arterial spin labeling (ASL) perfusion MRI, provides a completely noninvasive means to measure quantitative CBF within the brain [34]. It has been shown to be effective in detecting decreased perfusion in grey matter [15, 16, 17] and MS severity-related brain perfusion changes elicited by a psychological stress task [35]. Moreover, there has been additional data published showing a correlation between decreased perfusion and more severe functional disability [36, 37]. As ASL represents a non-invasive neuroimaging modality for quantifying CBF, it may be highly useful for the clinical evaluation of MS patients, as well as to follow them through various interventions designed to improve brain function and reduce the impact of the MS. Thus, our goal is to investigate changes in CBF in MS patients before and after receiving NAC.

## 2 Materials and methods

### 2.1 Overview of study

Written informed consent, approved by the Institutional Review Board of Thomas Jefferson University, was obtained from all subjects and the study was registered on clinicaltrials.gov with the following identifier: NCT03032601. Subjects were recruited from local neurology offices and also from the local community through meetings with MS support groups in the area. To be enrolled, subjects had to meet the standard clinical diagnosis of either relapsing remitting or progressive MS based upon the 2010 revision to the McDonald criteria [38] along with the following inclusion criteria: Age 21–80 years old and on stable medication regimen for at least one month. Patients were excluded if they had any of the following: any structural intracranial abnormalities that would prevent the analysis software from performing accurately (this does not include the typical white matter lesions found in MS patients); and any medical, neurological, or psychiatric disorder that could reasonably be expected to interfere with the assessment of MS symptoms, or with any of the study assessments including the resting ASL imaging to measure CBF (e.g. active cardiovascular disease, liver disease, movement disorders, stroke, active thyroid disease, major depression, bipolar disorder, and history of acetaminophen overdose). In addition, NAC is known to worsen asthma so a history of moderate to severe asthma was included as part of the exclusion criteria. There are only a few medications specifically known to interfere with NAC such as charcoal, ifosfamide, and inhaled insulin and these were excluded. In all, there were 23 MS subjects (17 relapsing remitting and 6 primary progressive; with a mean age of 53.4 ± 11.8 years) that qualified for the study and underwent an initial resting ASL MRI imaging as part of a PET-MRI scan. The PET scan was performed with FDG and this data was presented in a recently published article [31]. Briefly, FDG PET scans were obtained using standard technique before and after the NAC or waitlist condition. MIMNeuro software was used to obtain relative glucose metabolic activity in selected regions and comparisons were made between the pre and post intervention scans for the NAC and control groups. Of these subjects none had symptoms of relapse withing six months of entering the study and there were no relapses experienced during the study.

### 2.2 NAC intervention

Subjects were randomized using a permuted block method (using a 1:1 ratio with sealed envelopes for the allocation) either to receive intravenous plus oral NAC or to be placed in the waitlist control condition. Subjects were not blinded so they were aware that they were in one group or the other. A Fisher exact test was used to determine if there were significant baseline differences in variables such as diagnosis, medication use, or severity of disease based on the Expanded Disability Status Scale (EDSS). Subjects were also evaluated pre and post intervention with the Mental Health Inventory and the Perceived Deficits Questionnaire. These questionnaires ask patients about a variety of health problems that might be related to their MS diagnosis, for which we focused on the cognitive function and attention/concentration subscores respectively [39, 40]. These clinical questionnaires were statistically analyzed separately using linear mixed effects (LME) models with random patient effects. The fixed effects considered include age, gender, ethnicity, treatment group, timing (pre-therapy and post-therapy), as well as the interaction of treatment group and timing. For two months both groups continued their current standard of care management for MS, with the experimental group receiving NAC in both oral and intravenous form during that same period. NAC was obtained from the Jefferson Pharmacy as Acetadote (Cumberland Pharmaceuticals). Pharmaceutical grade NAC is an intravenous (IV) medication most commonly used for the treatment of acetaminophen overdose. Doses of NAC were prepared for each patient by a trained study nurse. The dose was 50 mg/kg mixed into 200ml of D5W infused over approximately 1 h 1x per week. Subjects additionally took 500mg NAC tablets 2x per day on the days that they did not receive the IV NAC. This was continued until presenting for the second MRI scan.

### 2.3 MRI acquisition, analysis, and statistics

#### 2.3.1 MRI acquisition

Participants first received the baseline MRI, including a high-resolution structural scan. A T1 MPRAGE sequence was used to perform this scan. The main purpose of acquiring structural image is to better examine cortical and subcortical volumes. This was followed by a resting state ASL scan to assess CBF in the different regions of the brain. The scans were acquired axially using a Siemens mMR 3T PET-MRI scanner with a 12 channel head coil. They were acquired at two time points; Pre-intervention and Post-intervention. The T1-weighted imaging parameters are as follows: TR = 1600 ms, TE = 2.46 ms, FOV = 252 mm × 252mm, voxel size = 0.5 × 0.5 × 1 mm3, slice thickness = 1 mm, and number of slices = 176. The Echo Planar Imaging (EPI) sequence parameters used for resting state ASL (ep2d_pasl) scans are as follows: TR = 2500ms, TE = 11ms, FOV = 192 mm × 192 mm, voxel size = 3 × 3 × 6 mm3. An asymmetrical pulsed ASL sequence (PICORE Q2T) [41] was utilized with the following parameters: 16 slices acquired in ascending order, slice thickness = 6 mm, Bolus Duration = 1675 ms, Inversion/Delay Time = 1800 ms, Labeling time = 700ms and labeling pulse flip angle = 90°. 119 volumes were acquired, with the 1st volume containing the M0 image and the remaining volumes containing 59 pairs of control/labelled images.

#### 2.3.2 MRI image processing

Preprocessing and statistical analysis was performed on all subjects using Matlab (ver. R2018a) using the standard steps that are incorporated as part of a validated SPM12 based ASL perfusion MRI data processing toolbox, ASLtbx [42]. The processing steps are as follows:1) Realignment: Rigid body spatial transformation was applied to the PASL control and label images in order to remove motion artifacts in the PASL time-series; 2) Co-Registration: the motion corrected PASL images and the M0 image were co-registered to the structural T1 space; 3) Smoothing: The PASL images were smoothed before CBF calculation to prevent noise propagation and to improve signal to noise ratio (SNR). A 6-mm full-width at half maximum (FWHM) Gaussian kernel was used; 4) Cerebral Blood Flow Calculation: Perfusion difference images were generated and the mean whole brain cerebral blood flow (ml/100 g/min) was calculated for each subject by pairwise control-label subtraction using the established one compartment model utilized by the ASLtoolbox [34, 43]; 5) Segmentation: the anatomical images were divided into individual tissue types (grey matter, white matter and CSF) for use in the outlier cleaning step. The high-resolution structural image (T1-MPRAGE) were used and compared to prior established tissue probability maps generated in SPM; 6) Outlier cleaning: Slice wise outlier cleaning was applied to the perfusion difference images produced in step 4 and utilized the segmentations produced in step 5 [44]; 7) Normalization: deformations produced from the SPM12 segmentation implemented in step 5 were applied to the outlier cleaned CBF maps to transform the functional data into the standard Montreal Neurological Image (MNI) space [45].

#### 2.3.3 Whole brain CBF quantitative analysis

The mean whole brain CBF values for each subject (generated in step 5 above) at the pre and the post scan (for both the NAC group and the control group) were calculated. The pre-scan whole brain values were tested for normal distribution using standard kurtosis and skewness calculation since these represented the baseline results for all MS patients. Then the difference in CBF between the post scan and the pre scan were calculated for each subject. Using these values, the average change in CBF in the treatment group and the control group was found and compared using ANOVA.

#### 2.3.4 Voxel level CBF comparison

Using SPM12 software, statistical analyses of the normalized, smoothed, outlier corrected perfusion images were performed in order to assess group differences in resting state cerebral blood flow between the control group and the treatment group. In order to find the effect of NAC treatment on CBF, the treatment and the control group were compared using a 2 × 2 full factorial ANOVA design, with condition (control and treatment) and time (pre and post) as the 2 factors utilized. The voxel based analysis was done on the whole brain, not predefined ROIs. Using a T-contrast reflecting the mean difference between the two groups and the variation within them in, the regions that survived FDR correction for multiple comparisons with a P value < 0.001 and cluster level >10 voxels were identified. These regions represent the change in CBF after 2 months of treatment specifically seen in the treatment group in comparison to the control group. We also explored whether there were any regions that correlated with changes in subjective measures of cognition and attention using linear regression analysis.

## 3 Results

### 3.1 Clinical and demographic sample characteristics

**Figure 1:**
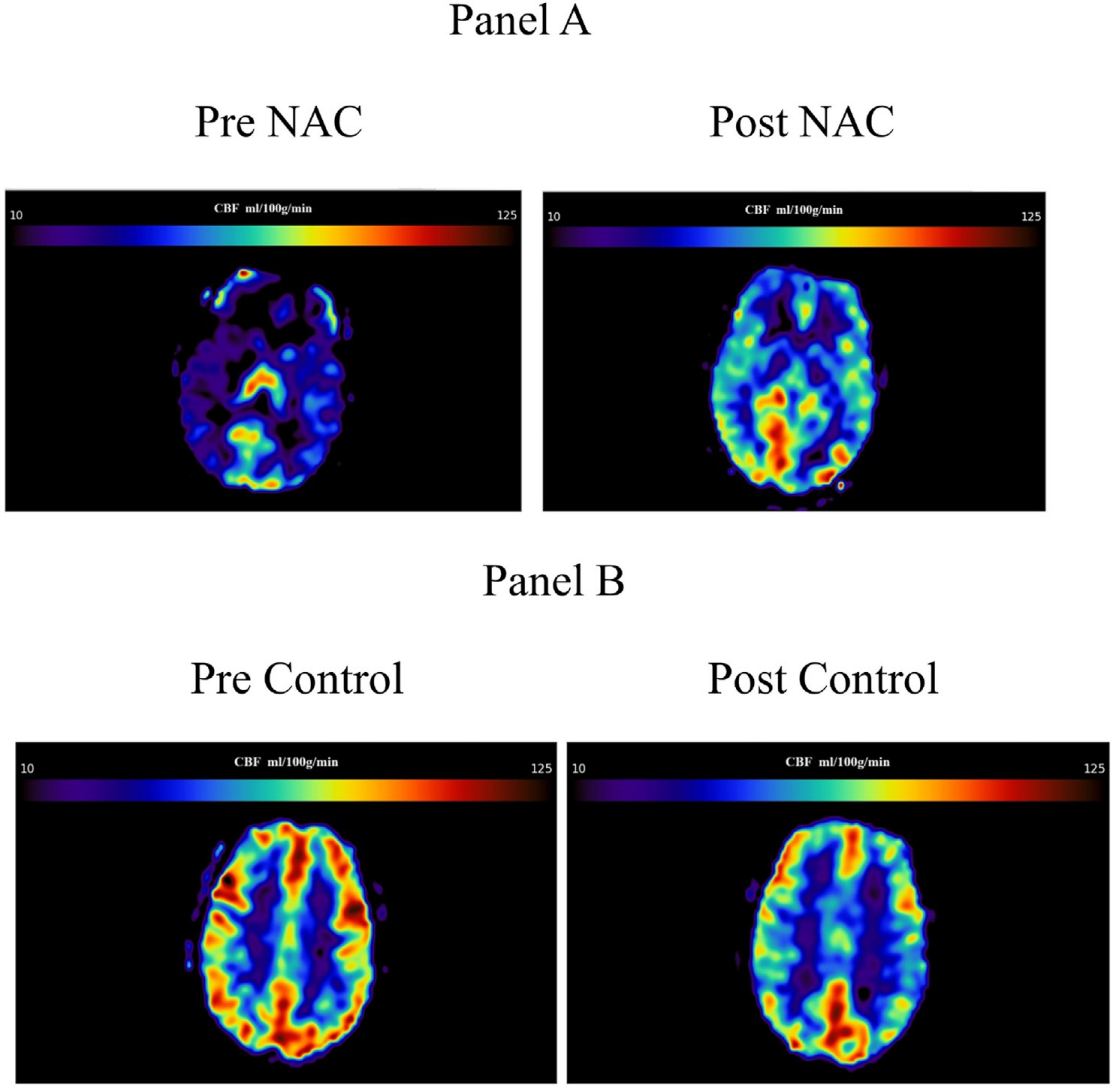
Panel A (top row) shows the comparison of the pre (left) and post (right) scan of an MS patient in the NAC group demonstrating a marked increase in global CBF after two months of receiving NAC. Panel B (bottom row) shows the comparison of the pre (left) and post (right) scan of a control patient revealing a decrease in global CBF during the waitlist period.

Table 1 shows the baseline clinical characteristics for all subjects including type of MS, use of MS medications, and EDSS score. There were no significant differences in these variables between the NAC and control groups. Table 1 (see also Figure 1) also presents the global cerebral blood flow changes between the NAC and control groups. There were significant improvements in the MHI cognition scores in the NAC group compared to controls (NAC increased or improved by a mean of 8.611 while control group decreased by a mean of 3.056; p < 0.05) and the PDQ attention scores (NAC decreased or improved by a mean of 1.417 while the control group increased 0.917; p < 0.05). It should be noted that these results have previously been reported [31].

**Table 1:** Demographic information regarding subject number, diagnosis, medication use and EDSS score. In addition, the pre and post intervention whole brain mean CBF is provided along with the difference.

Subject (Dx)	Treatment Group	MS Medications	EDSS	Pre Group Whole Brain Mean CBF ml/100 g/min	Post Group Whole Brain Mean CBF ml/100 g/min	Difference in CBF ml/100 g/min (Post treatment - Pre treatment)
MS001(RR)	NAC	None	3.0	36.19	48.78	12.59
MS002(RR)	NAC	None	4.0	62.25	64.86	2.61
MS003(RR)	NAC	Dalfampridine, Dimethyl fumarate	3.5	77.44	92.82	15.38
MS004(RR)	NAC	Interferon beta-1a	3.0	18.04	49.11	31.07
MS005(P)	NAC	Fingolimod	2.0	23.82	35.74	11.92
MS006(RR)	NAC	Interferon beta-1a	3.0	34.36	48.70	14.34
MS007(P)	NAC	Ocrelizumab	3.5	62.63	59.91	-2.72
MS008(RR)	NAC	teriflunomide	3.5	46.54	52.72	6.18
MS009(RR)	NAC	teriflunomide	4.0	33.53	47.03	13.50
MS010(RR)	NAC	Fingolimod	2.5	76.31	82.48	6.17
MS011(RR)	NAC	Interferon beta-1a	2.0	56.06	42.16	-13.90
MS001(P)	Control	None	4.5	59.04	37.09	-21.95
MS002(RR)	Control	Dalfampridine	5.0	75.81	55.60	-20.21
MS003(RR)	Control	None	2.0	38.18	30.21	-7.97
MS004(P)	Control	Dalfampridine Ocrelizumab	4.0	25.12	48.65	23.53
MS005(RR)	Control	Dalfampridine, Dimethyl fumarate	4.0	65.27	56.19	-9.08
MS006(RR)	Control	interferon beta-1a	3.0	83.03	90.98	7.95
MS007(P)	Control	Dimethyl fumarate	3.5	59.96	55.86	-4.10
MS008(P)	Control	Dalfampridine	2.0	85.69	73.89	-11.80
MS009(RR)	Control	Ocrelizumab	6.0	51.08	52.51	1.43
MS010(RR)	Control	teriflunomide	3.0	83.35	71.66	-11.69
MS011(RR)	Control	Natalizumab	2.0	110.54	89.17	-21.37
MS012(RR)	Control	Fingolimod	2.0	42.38	47.59	5.21

### 3.2 CBF analysis results

The CBF analysis of the brain demonstrated significant differences between the pre and post scans for the NAC versus the control group, however, there were no significant differences in the baseline or pre treatment scans between the two groups. Further, the pre-treatment whole brain scan data met criteria for uniformly distributed data with a kurtosis of -0.38 and skewness of 0.27. The NAC group had a mean whole brain increase in CBF of 8.8 ± 11.5 ml/100 g/min while the waitlist control group had a mean whole brain decrease in CBF of 5.8 ± 13.5 ml/100 g/min. ANOVA analysis revealed that these are significant changes both within group for the NAC intervention (within group comparison was significant with a p = 0.01) and when the NAC group was compared to the control group (across group comparison was significant with a p = 0.006).

Exploring individual regions, we found the following areas (see Table 2) were significantly increased (threshold set at p = 0.001) in the NAC group post treatment compared to the controls. The voxel-wise results are based on analysis of the whole brain, not predefined ROIs and the regions that survived FDR correction for multiple comparisons after the whole brain voxel based analysis are presented in Table 2. Specifically, there were significant increases in the pons, midbrain, left superior temporal lobe, left hippocampus/parahippocampus, left frontal lobe, left thalamus, and right temporal lobe/hippocampus. There was also significantly reduced blood flow in the right middle frontal gyrus. In comparison, our previously reported FDG PET data showed significant increases in metabolism in the caudate, inferior frontal gyrus, lateral temporal gyrus, and middle temporal gyrus [31]. No correlations were found between the changes in cerebral blood flow and subjective measures of cognition or attention even though these were significantly improved in the NAC group.

**Table 2:** Comparison of brain region differences in the NAC group compared to the control group between the pre and post scan. All regions represent significant differences between the NAC and control group with a p < 0.001 FDR corrected and a peak T-value.

Structure	Peak MNI Coordinate	Peak Intensity (T-Value)	Cluster Size (voxels)
Pons	0 -32 -30	3.67	11
Midbrain	14 -16 -12	3.82	26
L Superior Temporal Lobe	-36 0 16	4.24	16
L Hippocampus/Parahippocampus	-24 -38 -2	3.67	13
L Frontal Lobe	-38 -4 20	4.01	10
L Thalamus	-24 -22 8	4.53	56
R Middle Frontal Gyrus	42 56 8	-5.38	53
R Temporal/Hippocampus	36 -42 2	3.56	13

## 4 Discussion

An important facet of the pathophysiology of MS revolves around the induction of oxidative stress. It has been shown that oxidative stress in MS leads to numerous deficits such as damage to the brain endothelium, blood brain barrier, and dysfunction of mitochondria [46]. Other studies have also provided complementary data through observing an increase in oxidative stress markers in MS patients [23, 47]. Glutathione, an endogenous reducing agent, serves as antioxidant that aids in protecting neurons against oxidative damage. Additional data suggest that Glutathione levels are reduced in CNS of MS patients [8, 48, 49]. Similar reductions were also found in the periphery [50]. This reduction in glutathione along with its inherent limited supply within the brain predisposes MS patients to significant oxidative stress [51]. The accumulation of oxidative damage eventually overwhelms the brain's inherent cellular antioxidant mechanisms leading to dysfunctional and cell death. Although current therapies for MS are effective disease modification, interventions addressing the chronic oxidative damage may be effective in improving global brain function and alleviating neurological symptoms.

While numerous studies have investigated the effects of NAC in EAE rodents, we investigated the effects of NAC in human patient CBF. We hypothesized that by reducing oxidative stress with NAC administration, the neurons under duress from the MS pathophysiology might be able to recover function and subsequently blood flow to the affected regions. While the present study found changes in CBF based on ASL data, we cannot infer in a strict sense that the observed longitudinal perfusion changes reflect alterations/reductions in oxidative stress (especially given that a single ROI also showed a decrease of perfusion across time). A reduction in oxidative stress is one of several possible mechanisms underlying this observation and other possibilities include the ability of NAC to inhibit other inflammatory markers or to stimulate neuronal differentiation and neuritogenesis [52]. NAC might also have a direct effect on the vasculature leading to altered CBF (although there are no clear reports of such an effect in the literature). In a previous study we conducted, we were able to observe an increase in cerebral glucose metabolism in certain regions of the brain [31]. NAC presents itself as a promising therapeutic in regards to reducing oxidative damage in the brain. Moreover, it is available both as an oral supplement and as an intravenous agent. While NAC is readily available, there is still conflicting evidence in regard to NAC administration and elevation of glutathione levels in the brain. Further, it is unclear whether these changes in glutathione lead to substantial changes in cerebral blood flow and clinical scores. Studies investigating oral NAC absorption alone was not only variable but also low at (6–10%) [53, 54]. Some exercise studies have shown oral NAC to be effective in enhancing serum glutathione levels [48, 55]. Further, a MRS study using high doses of oral NAC (600 mg) did not find significant increases in glutathione in the brain [56]. Conversely, intravenous NAC administration increased brain glutathione levels [57]. Therefore, we decided to use a combination of both oral and intravenous administration in an effort to accommodate the logistical difficulties in receiving an injection multiple times a week while also addressing the low bioavailability of oral NAC. In previous studies involving a NAC intervention with Parkinson's and MS patients, we were able to observe physiological changes in the brain suggesting that this combination is sufficient in elevating and maintaining NAC levels in the brain [31, 58].

Our current study utilized the same combination of oral and IV NAC intervention in treating patients with relapse-remitting or primary progressive MS. Consequently, the results suggest significant changes in cerebral blood flow to a variety of brain regions. First, there was a global increase in mean CBF in the NAC intervention group in contrast to a global decrease in the control group. It is not completely clear the basis for the decrease in global CBF in the control group. It could represent some degree of acclimatization to the study procedures reducing anxiety during the imaging procedure, or it might represent some progressive neuronal dysfunction as the result of the disease process. Future studies could help confirm such a finding and attempt to determine its cause.

Interestingly, certain brain regions experienced an increase while others experienced a decrease. There were increases in CBF in the left superior temporal lobe, hippocampus, thalamus, and frontal lobe, as well as the right temporal/hippocampal region. There was decreased perfusion to the right middle frontal gyrus. This highlights the notion that NAC does not have a uniform effect on the brain but appears to target specific regions that are affected in MS. It is possible that some reductions might be beneficial if they result from increased inhibition that is required to maintain appropriate neuronal balance within the brain, especially with regard to motor activity [59].

To our knowledge, this is the first research study that has specifically evaluated the effect of NAC in patients with MS using resting CBF data. In a review study done by Tahedl et al. [60] the group investigated therapeutic intervention outcome on MS patients. Although most of these works used task-based CBF, they support the ability of such an approach as reliable method to monitor the improvement of the disease. In another study by Cader et al. [61], the study's aim was assessing the effect of administrating rivastigmine and domperidone as cholinesterase inhibitors on cognitive function of 15 MS patients using Stroop task and an N-back task. Task based CBF analysis of the N-back task in patients who took rivastigmine showed increase connectivity in the brain regions associated with cognition (i.e. prefrontal cortex). Patient group who took both medications, showed enhanced execution of Stroop task which was also associated with increased task based CBF in the prefrontal and parietal areas.

Importantly, areas of the brain with altered CBF observed in the current study, such as parts of the frontal and temporal lobe, also had increased metabolic activity. Thus, there appears to be an association between changes in CBF and cerebral glucose metabolism. This could be an important finding regarding the effect of NAC on brain processes as well as suggesting that future research consider combined imaging as a way of better understanding the effects of other therapeutic interventions in MS patients. It is also important to note that since areas such as the temporal and frontal lobe support cognitive processing and attention, the findings of increased CBF and increased glucose metabolism indicate a mechanism by which NAC might support these cognitive processes which we reported previously [31]. However, we were not able to detect significant correlations between regional changes in CBF and subjective measures of cognition or attention due both to the small sample size, the variability of measures, and the problem of multiple comparisons. Additional analyses in a larger sample size that more directly compare CBF and cerebral glucose metabolism might be beneficial for better determining this relationship.

This finding also supports the overall hypothesis that NAC may affect CBF in neurons initially under oxidative stress resulting in improved function and improvement in symptoms. This is also a potentially important finding since impaired cognition is a significant problem among patients with MS with between 40-70% of patients affected [19, 62]. Memory, information processing speed, and attention and executive function are cognitive domains that are most common in MS patients [63]. Cognitive impairment also has a significant effect on the quality of life of MS patients [64]. The pathophysiology of cognitive impairment in MS patients is multifactorial and includes effects of the disease itself, medications, mood problems, fatigue, and other factors. Neuroimaging correlates of cognitive impairment in MS patients include the degree of white matter lesions characteristic of MS, brain atrophy, and even abnormalities in otherwise normal appearing brain tissue [65]. There has been only limited studies evaluating resting CBF in relation to cognitive impairment in MS patients probing the correlation of cognition and resting state CBF in MS cohort, Cruz-Gumez [66] and the team, reported decreased CBF in default mode network, frontoparietal (both left and right) and salience regions of the brain. These regions were reported as the result of analysis in cognitively impaired patients. Another study by Louapre et al. [67] revealed that CBF was increased in attention related networks in cognitively preserved MS patients compared to control group. Whereas decreased connectivity in the default mode network and attentional networks was observed comparing cognitively impaired and preserved group. Thus, the findings from the current study are consistent with the above-mentioned studies and suggest that NAC might alter CBF in areas of the brain, such as the frontal and temporal lobes, that support improvements in cognitive function.

Although we observed significant changes in CBF in MS patients receiving NAC, there are several important limitations to our study. The study's sample size is small as we were limited by the costs of imaging and weekly NAC administration. Based on the mean and standard deviation for the whole brain CBF values, as well as the observed effect size, we would anticipate requiring at least 30 subjects per intervention group for future studies. In spite of this small sample, we were able to observe significant changes in CBF associated with NAC administration. It is noteworthy that while we included a control group, we did not perform a blinded study through giving control MS patients an IV placebo. Thus, it is plausible that the changes seen in CBF could be resultant, in part, due to a placebo effect. We opted against a blinded study as there were significant ethical concerns raised in giving MS patients IV placebo, and thus we opted for a waitlist control. Thus, it is possible then that the findings might be attributable to placebo effects although any physiological changes observed are expected to more likely reflect specific effects of the NAC, but future studies comparing NAC to a placebo injection in a blinded manner could elucidate any potential disparities. It would also be pertinent to collect a measurement of either NAC or glutathione as it is still unclear whether our intervention protocol elicits an increase of either molecule in the brain. We could acquire these objective measures through either a direct collection of CSF or through MR spectroscopy. Furthermore, it would be worthwhile to observe an extended study of NAC administration to observe any significant changes namely in clinical functionality and disease progression. Additional indirect measures could also include markers of oxidative stress to help provide further evidence to support the role of NAC in protecting neurons against oxidative damage. In regards to the changes seen in cognition and attention, we relied on self-reported questionnaires instead of objective neuropsychological measures. There is substantial evidence to suggest that self-reported and individual perception of cognition and attention can be affected by a myriad of factors such as stress, depression, sleep, and fatigue [68, 69, 70, 71]. We were able to observe slight improvements in these categories for patients, however, no results were statistically significant. It is our aim to use more objective measures of cognitive function such as specific neuropsychological testing in future studies with a larger number of subjects to further evaluate NAC's specific effects in the realm of patient neuropsychology.

## 5 Conclusion

The overall results of the present study reveal significantly increased whole brain CBF in MS patients after receiving two months of NAC compared to controls. Further, specific regions such as the midbrain, pons, and parts of the temporal and frontal lobe also had significant increases in CBF in the NAC group. Future, larger scale studies will be needed in order to determine if the findings in this initial study are corroborated and also correlated with improvements in specific symptoms and clinical markers.

## Declarations

### Author contribution statement

Shiva Shahrampour, Justin Heholt, Andrew Wang, Mahdi Alizadeh: Performed the experiments; Analyzed and interpreted the data; Wrote the paper.

Faezeh Vedaei: Analyzed and interpreted the data; Wrote the paper.

Feroze B. Mohamed, Andrew B. Newberg, Daniel A. Monti: Conceived and designed the experiments; Performed the experiments; Analyzed and interpreted the data; Contributed reagents, materials, analysis tools or data; Wrote the paper.

Ze Wang: Conceived and designed the experiments; Contributed reagents, materials, analysis tools or data; Wrote the paper.

George Zabrecky, Anthony J. Bazzan: Conceived and designed the experiments; Analyzed and interpreted the data; Wrote the paper.

Nancy Wintering, Thomas P. Leist: Conceived and designed the experiments; Performed the experiments; Analyzed and interpreted the data; Wrote the paper.

### Funding statement

This work was supported by a grant from the Adolph Coors Foundation.

### Data availability statement

Data will be made available on request.

### Declaration of interests statement

The authors declare no conflict of interest.

### Additional information

The clinical trial described in this paper was registered at clinicaltrials.gov under the registration number NCT03032601.
